# Current State of the Art in Ventricle Tissue Engineering

**DOI:** 10.3389/fcvm.2020.591581

**Published:** 2020-11-03

**Authors:** Ravi K. Birla

**Affiliations:** BIOLIFE4D, Houston, TX, United States

**Keywords:** bioengineering, bioreactors, patches, stem cells, electrical stimulation

## Abstract

The field of ventricle tissue engineering is focused on bioengineering highly functioning left ventricles that can be used as model systems for basic cardiology research and for cardiotoxicity testing. In this article, we review the current state of the art in the field of ventricle tissue engineering and discuss different strategies that have been used to bioengineer ventricles. Based on this body of literature, there are now common themes in the field that provide guidance for future directives, also presented in this article.

## Overview of Cardiac Tissue Engineering

According to the American Heart Association, Heart Disease and Stroke Statistics update 2020 (1), the most recent year available at the time of this publication, cardiovascular disorders accounted for 859,125 deaths in the US out of a total of 2,813,503 registered deaths for the year 2017 (1). Cardiovascular disorders come at a very high cost, estimated to be $1.1 trillion by the year 2035, with direct medical cost estimated to reach $748.7 billion and indirect cost estimated to be $368 billion (1). Given the severity of cardiovascular disorders in the USA, there is an urgent need to develop novel and innovative therapies to help these patients. Cardiac tissue engineering is one such example, a field in which scientists and researchers are working rigorously to develop innovative technologies to help patients with cardiovascular disorders in need of these lifesaving therapies (2, 3).

Research in the field of tissue engineering is focused on fabricating tissue and organs that can be used for clinical transplantation. One of the first publications describing engineering of functional tissue was in 1988, when it was shown that primary hepatocytes isolated from rodent were cultured in three-dimensional matrices fabricated using three different polymers, polyglactin, polyorthoester, and polyanhydride (4). The primary hepatocytes were shown to maintain viability, function, and engraftment within the three-dimensional matrices. This work was seminal as it was the first time that primary cells were cultured within three-dimensional matrices to form functional tissue, the basis of tissue engineering as a scientific discipline.

**Figure 1:**
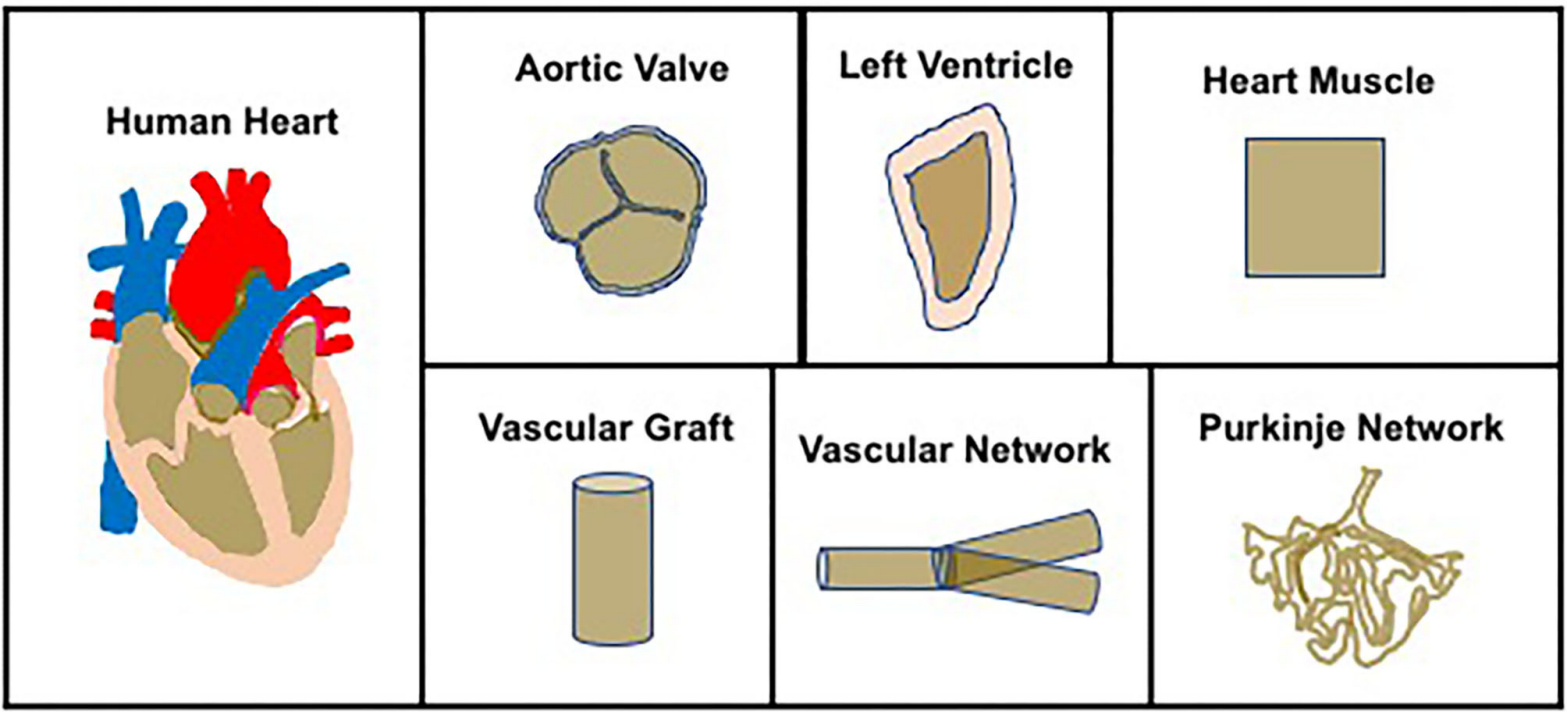
Overview of cardiac tissue engineering—The field of cardiac tissue engineering is focused on bioengineering whole hearts or components of the heart, to include aortic valve, left ventricles, heart muscle, vascular grafts, vascular networks, and Purkinje networks.

The field of cardiac tissue engineering includes heart muscle tissue (2, 3), aortic valves (5), vascular grafts (6–8), vascular networks (9, 10) and Purkinje networks, left ventricles (11), and whole hearts (12) (Figure 1). While heart muscle tissue engineering is now an established field (3), the same is not true for ventricle bioengineering, a niche field and the focus on this review article.

## Process to Bioengineer Ventricles

**Figure 2:**
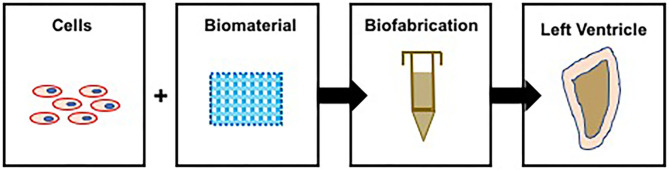
Process to bioengineer ventricles—contractile CMs are coupled with biomatrices and used to bioprint functional ventricles. Note: in this illustration, bioprinting is used to illustrate the process to bioengineer ventricles.

There is a large body of literature describing methods to bioengineer cardiovascular tissue constructs (13–17), based on which a methodological process has now evolved (Figure 2). The first step in the process is cell sourcing, generating a large number of contractile cardiomyocytes (CMs). Neonatal ventricular rat myocytes (NVRMs) are used for initial proof-of-concept studies and model development and validation efforts, while induced pluripotentent stem cell (iPSC)-derived CMs are used for clinical studies. The second step involves biomaterial synthesis, which entails the development of novel biomaterials that can be used to simulate mammalian extracellular matrix (ECM). A large number of biomaterials have been used in the field, to include collagen type I, fibrin, gelatin, alginate, and chitosan, to name a few (18). The third step involves coupling contractile CMs with novel biomaterials to generate functional ventricles, in other words, develop novel fabrication methods to bioengineer functional ventricles. The fourth and final step is *in vitro* conditioning of bioengineered ventricles using bioreactors for coupled electromechanical stimulation.

## The Need for Bioengineered Ventricles

The notion to bioengineer cardiac patches is easy to understand as the potential applications to repair infarcted myocardium tissue are now well-established (18, 19). Similarly, the utility of vascular grafts, aortic and mitral valves, and whole hearts for potential therapeutic purposes is evident and well-described (19). However, the rationale to bioengineer ventricles is not as straightforward as there is no obvious therapeutic target for bioengineered ventricles. However, irrespective of the lack of a clear therapeutic target, there are many reasons to bioengineer ventricles. First and foremost, the holy grail of tissue engineering is to bioengineer a complete bioartificial and the experience gained in ventricle bioengineering is critical to accomplishing this goal. Second, bioengineered ventricles provide valuable tools to study fluid dynamics in an isolated *in vitro* system and can also be used to understand the role of pressure and volume overload and other complex physiological phenomena, oftentimes difficult to study using existing models. Third, a complete left ventricle that contains a hollow chamber, contractile heart muscle, and mitral valves, including outflow and inflow tracts, can be used to investigate and understand the interrelationship between all these tissue components during heart function, something that would not be possible using any other *in vitro* or *in vivo* model systems. Fourth, bioengineered ventricles can be used to study cardiotoxicity effects of pharmacological agents, thereby reducing the animal cost.

### Strategies to Bioengineer Functional Ventricles

**Figure 3:**
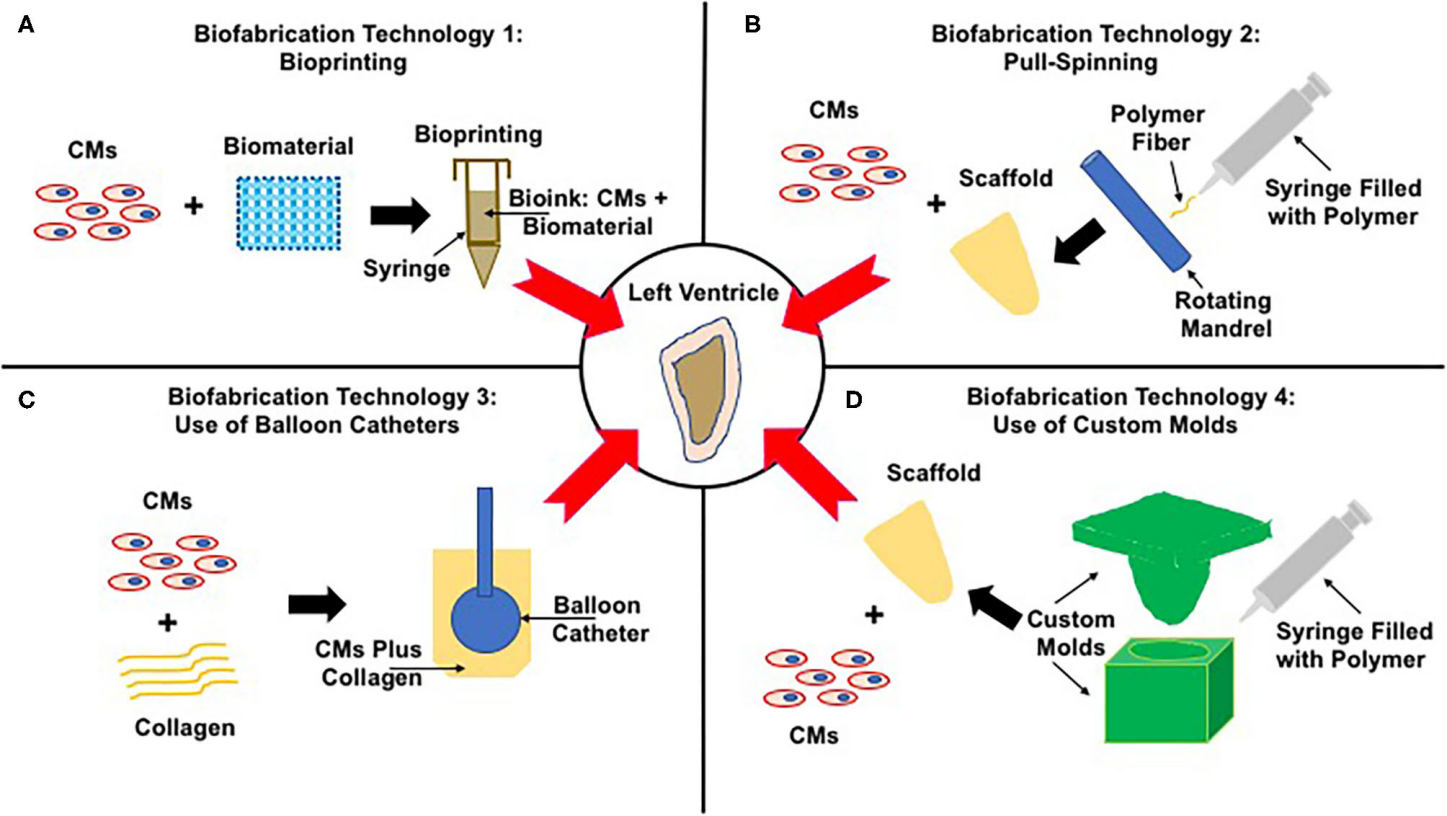
Strategies to bioengineer functional left ventricles. The center panel shows the end-goal of a functional left ventricle. Panels **(A–D)** illustrate four very different strategies to achieve this end goal. **(A)** Biofabrication technology 1: Bioprinting—contractile CMs are combined with a biomaterial to generate bioink, which is loaded into a printing syringe and used to bioprint ventricles. **(B)** Biofabrication technology 2: Pull-Spinning—the polymer is first loaded into a syringe and then extruded toward a rotating madrel that collects and shapes the polymer fibers to form ventricle scaffolds. These scaffolds are then populated with contractile CMs, yielding functional ventricles. **(C)** Biofabrication technology 3: Use of Balloon Catheter—primary CMs are mixed with collagen and seeded around the outer surface of a balloon catethar, a process that results in the fabrication of left ventricles. **(D)** Biofabrication technology 4: Use of custom molds—the polymer is loaded into the molds, the void space of which supports the fabrication of ventricle scaffolds, which are next seeded with contractile CMs supporting the formation of functional left ventricles.

There are several different established strategies in place to bioengineer ventricles, differing in the source of contractile CMs and biomaterial and biofabrication technology (Figure 3, Table 1). The major difference in these methods lies in the biofabrication technology, introduced here and discussed in detail in subsequent sections.

Biofabrication Technology 1: Bioprinting (Figure 3A, Table 1)—cells are combined with a biomaterial and loaded into a syringe, and a software program is used to guide the extrusion of the cell–biomaterial mixture (known as the bioink) to print a 3D structure, a ventricle in this case.
Biofabrication Technology 2: Pull-Spinning (Figure 3B, Table 1)—the biomaterial is ejected from a nozzle to a rotating collection device resulting in the formation of a ventricle scaffold, which is then populated with contractile CMs.
Biofabrication Technology 3: Use of Balloon Catheter (Figure 3C, Table 1)—contractile CMs coupled with a biomatrix and cast around the outer surface of a balloon catheter to generate hollow chambered ventricles.
Biofabrication Technology 4: Use of Custom Molds (Figure 3D, Table 1)—negative molds are first fabricated and then used to bioengineer ventricle scaffolds, which are then populated with contractile CMs.

**Table 1:** Methods to bioengineer functional ventricles.

**Year**	**Cells**	**Matrix**	**Fabrication method**	**Bioreactors**	**Valve**	**Pressure (mm Hg)**	**Electrical properties**	**Calcium transients**	**PV loops**	**Novelty**	**References**
2018	iPSCs-CMs and NVRMs	PCL and Gelatin	Pull spinning	Yes, mechanical loading	YES	0.05	NO	YES	YES	Use of pull spinning	(3)
2018	ESCs-CMs	Collagen type I	Balloon catheter	Yes, electrical stimulation	NO	0.09	YES	NO	YES	Use of catheters	(4)
2018	NVRMs	Chitosan	Custom molds	Yes, pulsatile fluid flow	YES	3.1	YES	NO	NO	2-stage cellularization strategy	(5–8)
2020	iPS-CMs	Collagen type I	Bioprinting	NO	NO	Not reported	NO	YES	NO	Use of FRESH technology	(2)

*iPSC, induced pluripotent stem cells; CMs, cardiomyocytes; ES, embryonic stem cells; NVRMs, neonatal ventricular rat myocytes*.

### Biofabrication Technology 1: Bioprinting

Bioprinting is a process by which cells are first combined with the biomaterial to form bioink, which is next loaded into a syringe, and using pneumatic pressure, the bioink is extruded from the syringe a single layer at a time. Multiple layers are extruded, often times 100 or more, each layer with a thickness of 100–500 microns, to form a complex 3D tissue and/or organ structure (19). Bioprinting is an established method and has been used extensively in cardiovascular tissue engineering (20–25). The primary advantage of bioprinting is the ability to regulate the spatial organization of different cell types relative to each other and relative to the ECM. Since all mammalian tissue and organs are composed of multiple cell types within complex ECM, bioprinting allows recapitulation of mammalian tissue/organ anatomy.

Bioprinting was successfully used to bioengineer functional left ventricles, using iPSC-derived CMs and type I collagen as the biomaterial (Figure 3A, Table 1) (11). One of the novel elements of this study was the use of a gelatin-based slurry to increase print fidelity. Traditional methods of bioprinting are based on extrusion of the bioink in air, a process that reduces print fidelity of soft biomaterials like gelatin, fibrin, alginate, and agarose. Rather than printing in air, printing directly into a support slurry provides support during the printing process, and once the print is complete, the support slurry is washed away through a temperature-dependent mechanism.

Using this process, a complete bioartificial left ventricle was bioprinted and shown to exhibit calcium transients with a calcium conduction velocity of 1.3 cm/s and served to establish function of the bioprinted ventricles (11). However, the ventricles were printed as open structures, and as a result, it was not possible to measure LV pressure, a critical determinant of ventricle function and the most obvious next step for this technology.

### Biofabrication Technology 2: Pull-Spinning

A recent study described the use of a novel fabrication method, known as pull-spinning, to bioengineer ventricles (Figure 3B, Table 1) (26). In this method, polycaprolactone (PCL) was mixed with gelatin, loaded into a syringe, ejected through a nozzle, and collected on a spinning mandrel to replicate the anatomical characteristics of the human left ventricle (26). The ventricle scaffold was populated with NVRM- or iPSC-derived CMs. Calcium transients were used to assess function, pressures of 0.05 mm Hg were noted, and pressure–volume relationships were demonstrated (26). These ventricles were then fitted with valves and cultured in a pulsatile flow bioreactor for mechanical conditioning (26). This was an outstanding study that clearly demonstrated the feasibility of bioengineering ventricles, including tri-leaflet valves and the use of bioreactors for mechanical loading of the bioengineered ventricles.

### Biofabrication Technology 3: Use of Balloon Catheters

A very interesting approach is the use of Foley balloon catheters to provide support for a cell-laden mixture of type I collagen supplemented with matrigel and CMs derived from human embryonic stem cells (ESCs) (Figure 3C, Table 1) (27). The entire structure, balloon catheter, and CMs were housed in a custom chamber with 2% agarose being used as the sacrificial material (27). The collagen and CM mixture was allowed to gel in place for 24 h in the presence of the sacrificial material, which was then removed and supported the formation of a hollow chamber ventricle structure (27). Electrical stimulation was used during the culture period (27). Ventricles that were bioengineered using this method were shown to generate left ventricle (LV) pressures of 1.2 mm H_2_O or 0.09 mm Hg (27).

### Biofabrication Technology 4: Use of Custom Molds

A series of four publications described a systematic and methodological process to bioengineer functional ventricles (Figure 3D, Table 1) (28–31). All studies were based on chitosan as the biomaterial and NVRMs were used as the cell source. The first study described the fabrication of an open ventricle mold, the second study described a novel 2-stage cellularization strategy, the third study described the use of bioreactors, and the fourth study included valves to complete the left ventricle model. These four studies are described in detail below.

Study 1: Development of an open ventricle—this study was focused on developing and validating the fabrication of ventricles and on material synthesis and characterization (30). Custom molds were machined to replicate the geometrical properties of human neonatal left ventricles, and chitosan was used as the biomaterial (30). A detailed material characterization was presented and included scanning electron microscopy, atomic force microscopy, and Fourier transform infrared for spectral analysis, all of which served to demonstrate the suitability of chitosan for ventricle bioengineering (30).
Study 2: 2-stage cellularization strategy—in previous studies, direct cell injection has been used to cellularize scaffolds, though this is known to result in low cell retention as a large number of cells are washed away (32). This remains a major challenge in the field of tissue engineering. This second study in this series addressed this problem and developed a novel 2-stage cellularization strategy to improve cell retention. Stage 1 consisted of direct injection of NVRMs within the ventricle scaffold. Subsequently, in stage 2, a highly contractile 3D cardiac patch was anchored on the outer surface of the cellularized ventricles. The contractile cardiac patch increased cell retention by providing a barrier function and also provided contractile support for the bioengineered ventricles (31).
Study 3: bioreactor conditioning—after developing the methodology to bioengineer ventricles and the development of a novel 2-stage cellularization strategy, the next step in the technology development was the fabrication of pulsatile flow bioreactors (28). Bioreactors were custom fabricated and were designed to mechanically load the bioengineered ventricles, designed to increase the contractile performance of the bioengineered ventricles. Culturing the ventricles in these custom bioreactors for 20 h resulted in a significant increase of pressure, from 0.2635 ± 0.1087 mm Hg to 3.1633 ± 0.3589 mm Hg (28).
Study 4: left ventricles with valves—In the fourth and final study, the model of the neonatal left ventricle was completed by the incorporation of a valve, also fabricated using chitosan (29). This resulted in the formation of a complete left ventricle, similar in form and function to human left ventricles and a truly outstanding accomplishment in the field of ventricle tissue engineering.

## Where Have We Been?

Our approach to answering this question is to explore at different strategies for the key components of the ventricle fabrication process, namely, fabrication technology, biomaterial, cell source, bioreactors, and functional assessment; see Table 1. The goal is to critically evaluate the current state of the art and provide a directive moving forward.

### Fabrication Technology

There have been four different methods to bioengineer ventricles (11, 26, 27, 29). Bioprinting relies upon a mechanical force to extrude bioink (11), pull-spinning relies upon a rotating mandrel to collect extruded fibers (26), use of balloon catheters relies upon an external support to bioengineer hollow ventricles (27), and custom molds cast the biomaterial in the form of a ventricle (29). All fabrication methods have proven successful in bioengineering functional ventricles. None has a clear advantage. A pre-requisite to the field of ventricle tissue engineering progressing is both refinement of existing fabrication methods and development of additional technology to support ventricle bioengineering.

### Biomaterials

The most broad area of ventricle bioengineering, with many different biomaterials being used [chitosan (29), type I collagen (11, 27), and a mixture of PCL and gelatin (26)]. As the field progresses, additional biomaterials will need to be developed that more closely match the mechanical and biological properties of mammalian ventricles and this continues to be an area of active investigation (18, 33).

### Cell Sourcing

The gold standard in the field is iPSC-derived CMs, as they provide an autologous source of contractile CMs. However, these cells are expensive to generate, limited in number, and functionally immature. NVRMs are abundant, inexpensive, and reproducible. An efficient interplay between NVRMs and iPSC-derived CMs will be critical for the advancement of ventricle tissue engineering, with the former being used for model development and validation studies and the latter being used for translational studies.

### Bioreactor Technology

The need for bioreactors in ventricle tissue engineering is well-established in the field, and recent publications have demonstrated the importance of electrical stimulation (27) and pulsatile fluid flow (28). However, customization by individual labs reduces comparison of results and efficient flow of information between individual researchers, a problem that can be solved by open-source sharing of bioreactor designs by independent Scientist in the field.

## Where Do We Need to Go?

While much progress has been made in the field of ventricle tissue engineering, a structured and methodological approach is needed moving forward. Here we provide guidance for the field of ventricle tissue engineering moving forward. Model development and optimization studies need to be conducted with NVRMs to achieve left ventricular pressures of 120 mm Hg coupled with parallel studies using iPSC-derived CMs for patient-specific disease models, for biomarker development and development of new investigational strategies. CMs derived from iPSCs are known to be immature, and the ability to develop maturation paradigms is critical for the field of ventricle tissue engineering and tissue engineering as a whole. Controlled electromechanical regimes coupled with chemical conditioning using growth factors and cytokines are required to achieve this. New biomaterial formulations continue to be developed and optimized, and existing tissue fabrication methods continue to be refined and adopted for ventricle bioengineering (34). A critical hurdle that needs to be overcome is the development of patent vasculature that can support the viability of contracting CMs. This is critical to supporting the metabolic activity of contracting cells, and in the absence of engineered vasculature, tissue-engineered constructs are limited in thickness.

Bioreactors need to be more consistent between labs to allow information exchange (35, 36), which in turn will accelerate development of ventricles for any given applications; sharing of software and code hardware components through open-source mechanisms will be required and will be a much-needed advancement in the field of ventricle tissue engineering.

Though challenges remain in the field, recent advances provide positive guidance and suggest that the ability to bridge the functional gap between bioengineered and human ventricles is achievable in the near future.

## Author Contributions

The author confirms being the sole contributor of this work and has approved it for publication.

## Conflict of Interest

RB is CSO of a Biotech Company that worked on bioprinting human hearts.
